# Internal limiting membrane detachment in acute central retinal artery occlusion: a novel prognostic sign seen on OCT

**DOI:** 10.1186/s40942-021-00323-7

**Published:** 2021-09-03

**Authors:** Ramesh Venkatesh, Chaitra Jayadev, Akhila Sridharan, Arpitha Pereira, Nikitha Gurram Reddy, Jophy Philip Cherry, Naresh Kumar Yadav, Jay Chhablani

**Affiliations:** 1 Dept. of Retina and Vitreous, Narayana Nethralaya, #121/C, 1st R Block, Chord road, Rajaji Nagar, Bengaluru, 560010 Karnataka India; 2 Medical Retina and Vitreoretinal Surgery, University of Pittsburgh School of Medicine, 203 Lothrop Street, Suite 800, Pittsburg, PA 15213 USA

**Keywords:** Central retinal artery occlusion, Internal limiting membrane detachment, Optical coherence tomography, Pathogenesis, Prognosis

## Abstract

**Background:**

To present a series of acute central retinal artery occlusion (CRAO) cases showing internal limiting membrane detachment (ILMD) on optical coherence tomography (OCT) and to describe the possible etiopathogenesis and outcomes associated with it.

**Methods:**

Demographic and OCT features of patients with acute CRAO were analysed retrospectively. OCT parameters noted were posterior vitreous opacities, ILMD, inner retinal layer stratification, hyperreflectivity and thickening, cystoid macular edema, neurosensory detachment. Eyes were grouped into Group (1) CRAO with ILMD; Group (2) CRAO with no ILMD.

**Results:**

A total of 28 eyes of acute CRAO who had undergone OCT scans at the time of the acute episode were identified. Out of these, ILMD was noted in 5 eyes. The study findings suggested that cases of acute CRAO with ILMD are associated with poor presenting visual acuity and have more severe signs of retinal hypoperfusion on OCT, like inner retinal thickening, inner retinal hyperreflectivity and loss of inner retinal layer stratification. Patients with ILMD have poor final visual acuity and thinning and atrophy or necrosis of the inner retinal layers.

**Conclusion:**

ILMD can occur in acute CRAO due to total retinal artery occlusion and severe retinal hypoperfusion. The presence of ILMD on OCT can be considered a sign of poor prognosis in cases of acute CRAO.

*Trial registration*: Not applicable.

## Background

Central retinal artery occlusion (CRAO) was first described by von Graefes in 1859 [1]. It is a disastrous ophthalmic emergency which presents with acute painless vision loss and carries a poor prognosis. Acute CRAO classically presents with retinal whitening, a cherry red spot and retinal vessel attenuation [2]. According to Hayreh, CRAO mainly affects the inner retinal layers [3]. In addition, different inner and outer retinal features of CRAO on optical coherence tomography (OCT) have also been described in literature [4]. The typical findings seen on the OCT include presence of inner retinal layer thickening, hyperreflectivity and loss of inner retinal layer stratification (which are directly related to the severity of occlusion) and retinal hypoperfusion [4]. No vitreoretinal interface changes have been reported immediately following the occlusion of the central retinal artery. The intraretinal hyperreflectivity of the acute phase gradually disappears leaving behind a thinner retina with atrophic inner layers [5]. On histopathology, there is inner ischemic retinal thinning of the nerve fibre layer, ganglion cell layer, inner plexiform layer and inner nuclear layer after occlusion of retinal circulation [6]. Considering the ischemic changes to the inner retinal layers, there is no affection of the ILM following CRAO. In this study, the authors present a series of acute CRAO cases showing a peculiar finding on OCT, which is described as the internal limiting membrane detachment (ILMD) and further venture to describe the possible etiopathogenesis and outcomes associated with the presence of ILMD in acute CRAO cases.

## Methods

This work was approved by our Institutional Review Board and Ethics Committee and respected the tenets of the Declaration of Helsinki. In this retrospective non-interventional study, analysis of optical coherence tomography (OCT) images in patients with acute CRAO identified through the institute’s internal database from April 2017 to March 2020 was included. The study was carried out at the Narayana Nethralaya eye hospital, India. An acute obstruction of central retinal artery was defined as recent history of sudden, painless loss of vision with presence of retinal thickening and whitening, retinal vessel attenuation and a cherry red spot at the macula. The medical records of all acute CRAO cases were reviewed and the following data were collected: age, gender, involved eye, corrected distance visual acuity at presentation and final visit, clinical features on retinal examination and follow-up duration. Patients with a history of ocular trauma, macular disease, vitreomacular interface disorders, severe non-proliferative or proliferative diabetic retinopathy, ocular surgery other than cataract surgery or high myopia were identified and excluded from the study. Such patients may have previous OCT findings that could include or lead to ILM detachment.

A high-resolution spectral-domain OCT scan was obtained using the Spectralis HRA OCT system (Heidelberg Engineering, Heidelberg, Germany) with 25-line horizontal volume scans covering the area centered on the fovea. The descriptive features which were noted on SD-OCT included the presence of posterior vitreous opacities, ILMD, inner retinal thickening, hyperreflectivity and loss retinal layer stratification, presence of cystoid macular edema and subretinal fluid during the acute phase and thinning and atrophy of the retinal layers during the chronic phase of the disease.

## Results

**Fig. 1:**
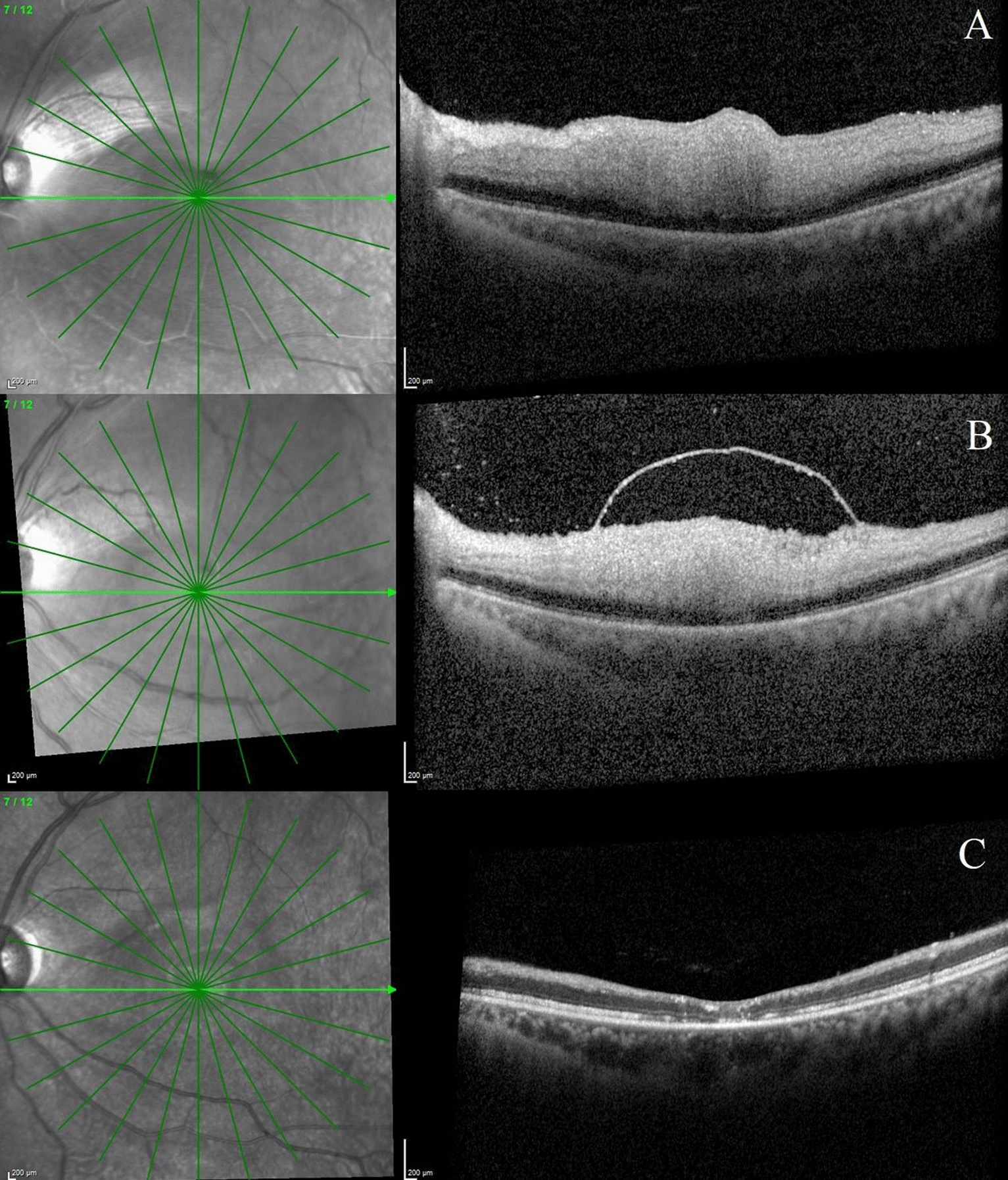
Optical coherence tomography scans of the left eye of Case 1 with central retinal artery occlusion and sparing of cilioretinal artery. **a** Left eye optical coherence tomography (OCT) scan at presentation showing inner retinal thickening, hyperreflectivity and loss of inner retinal layer stratification. **b** At 5 days post presentation, left eye OCT image shows presence of posterior vitreous opacities, internal limiting membrane detachment (ILMD), inner retinal thickening and hyperreflectivity and loss of inner retinal layer stratification. **c** At 4 weeks post presentation, there is inner retinal layer thinning and atrophy. The ILMD has resolved

**Fig. 2:**
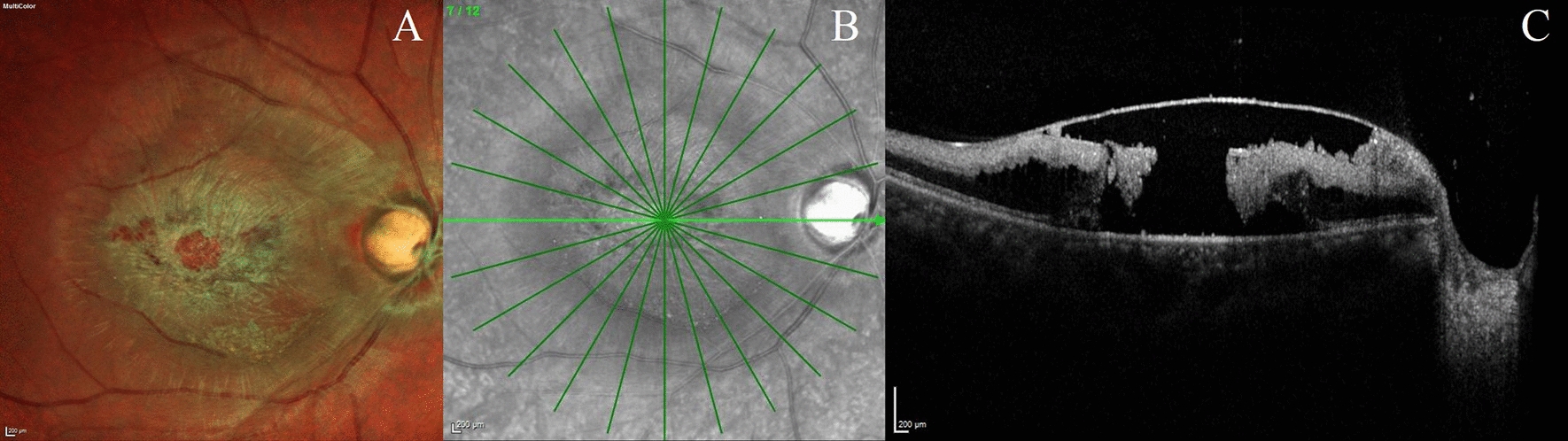
Multicolour image and optical coherence tomography image of the right eye of a 30-year-old man diagnosed with central retinal artery occlusion (Case 2). **a** Multicolour image of the right eye (Spectralis, Heidelberg Engineering) presenting to the retina clinic after 1-week of symptoms showed an optic nerve head a with high cup: disc ratio (0.8) with peripapillary whitening, retinal vessel attenuation, and a thin necrotic type of retina and macular hole. **b** The optical coherence tomography scan of the right eye shows hyperreflective spots at the posterior pole with internal limiting membrane detachment and a full thickness macular hole defect with hyperreflectivity of the inner retinal layers and degeneration and atrophy of the retinal layers

**Fig. 3:**
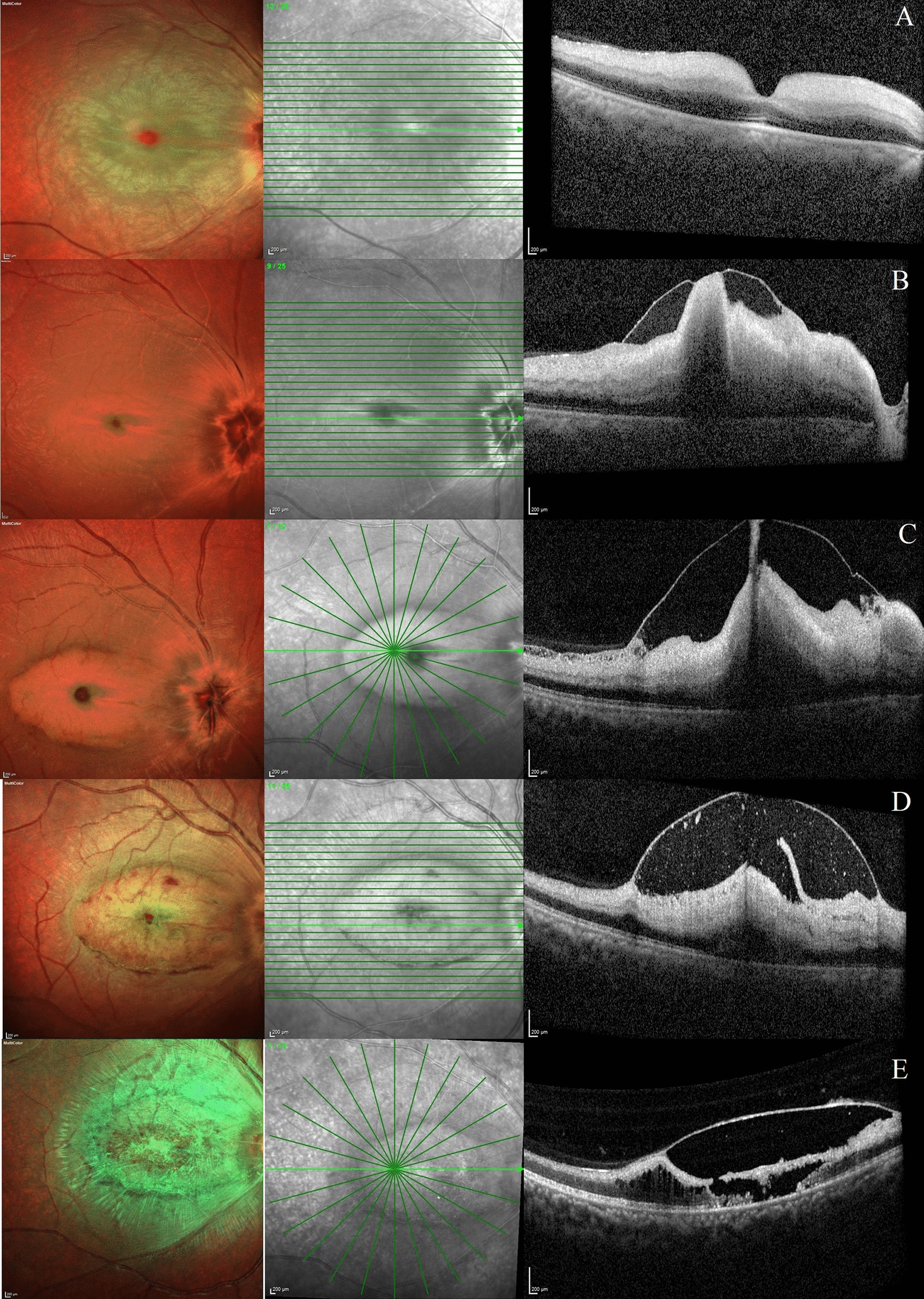
Evolution of internal limiting membrane detachment following acute central retinal artery occlusion (Case 4). **a**–**e** Fundus and optical coherence tomography scan of the right eye of a 25-year-old male who presented with sudden onset of decrease in vision secondary to central retinal artery occlusion. The images have been taken on day 0, 1, 3, 7 and 35 respectively

**Fig. 4:**
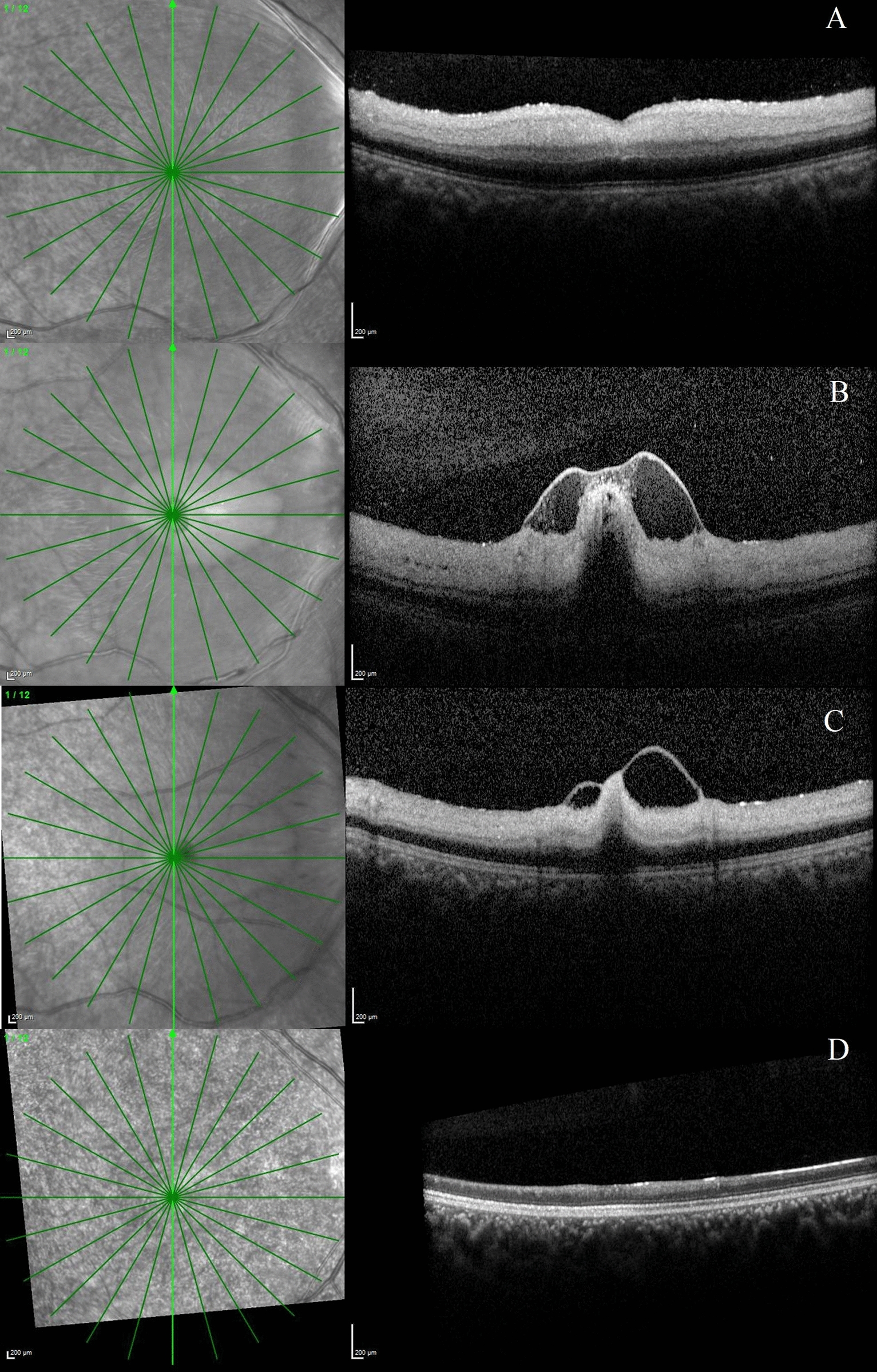
Sequential optical coherence tomography scans of the right eye in a patient with acute central retinal artery occlusion developing internal limiting membrane detachment (Case 5). **a** Optical coherence tomography (OCT) scans of the macula at presentation showing inner retinal thickening, hyperreflectivity and loss of retinal layer stratification. Immediate ocular massage with anterior chamber paracentesis was done and the patient was started on oral acetazolamide. **b** After 3 days of presentation, the OCT scan shows hyperreflective spots in the posterior vitreous, inner retinal thickening and hyperreflectivity, and loss of retinal layer stratification. Internal limiting membrane detachment was noted at the fovea. Hyperreflective dots and material were noted within the separated ILM space. **c** At 10 days post presentation, there is reduction in the retinal thickness, reduced hyperreflectivity and layering of the inner retinal layers is identified. **d** At the final visit, 4 weeks post presentation, the OCT scan shows total atrophy and thinning of the retinal layers with no improvement in visual acuity

A total of 28 eyes of acute CRAO who had undergone OCT scans at the time of the acute episode were identified. Out of these, ILMD was noted in 5 eyes and no ILMD cases were 23. Their clinical and OCT findings have been documented in detail in Table 1; Figs. 1, 2, 3 and 4. A comparison of the OCT findings between eyes with ILMD and eyes without ILMD in cases of acute CRAO are described in Table 2. The mean duration of symptoms in the ILMD group was 3.8 (1–7) days and in the non-ILMD group was 3.78 (1–10) days respectively. All the cases in the ILMD group presented with a visual acuity of ≤ 3/60. 16 (70%) eyes in the non-ILMD group presented with a visual acuity of ≤ 3/60. Two patients each were lost to follow-up in the ILM detachment group and non-ILM detachment group respectively. At the final visit, all the patients in the ILMD group had a poor visual acuity (range: perception of light (PL) present to PL absent) while only 44% (10 eyes) of the cases in the non-ILMD group had a final visual acuity of ≤ 3/60. In the ILMD group, restoration of the ILMD was noted in one eye while thinning and atrophy of the inner retinal layers was noted in 3 eyes. Retinal thinning and atrophy were noted in 21 eyes in the non-ILMD group at the final visit.

**Table 1:** Clinical and OCT features of patients with internal limiting membrane detachment (ILMD) following acute central retinal artery occlusion (CRAO)

No	Age/sex	Systemic disease	Affected eye	Duration of symptoms	IOP	VA at presentation	Fundus features	Clinical diagnosis	OCT features at the time of ILMD development	Time interval between presentation and ILMD	Ocular treatment given	Vision at final visit	OCT features at final visit	Follow-up duration
1	35/M	None	LE	1 Day	13	PL–ve	1) Retinal whitening 2) Cherry red spot 3) An island of normal retina temporal to the optic disc 4) Normal optic nerve head	CRAO with CLA sparing	1) Posterior vitreous opacities 2) Inner retinal thickening 3) Inner retinal opacification 4) Loss of retinal layer stratification 5) ILMD noted at the posterior pole with a few low intensity hyperreflective dots noted within the ILM detachment	5 days	1) Ocular massage 2) Anterior chamber paracentesis 3) Oral Acetazolamide 250 mg	PL–ve	Atrophy and thinning of the inner retinal layers with reattachment of ILMD	28
2	30/M	CVA	RE	7 days	12	PL–ve	1) Pale optic nerve head 2) Peripapillary whitening 3) Retinal vessel attenuation 4) A thin atrophic retina 5) Possible macular hole	CRAO with macular hole	1) Posterior vitreous opacities 2) Inner retinal thinning 3) ILMD noted at the posterior pole with a few hyperreflective dots noted within the ILMD 4) Full thickness macular hole defect	At presentation	1) Oral Acetazolamide 250 mg	–	–	–
3	69/F	None	LE	7 days	11	CF 1mt	1) Retinal opacification at the posterior pole 2) Cherry red spot 3) Retinal vessel attenuation	CRAO	1) Posterior vitreous opacities 2) Normal retinal thickness 3) Retinal opacification 4) Normal foveal contour 5) ILMD at the fovea	At presentation	Referred to internist	–	–	–
4	25/M	None	RE	3 h	16	PL + ve	1) Retinal whitening 2) Cherry red spot 3) Normal optic nerve head	CRAO	1) Posterior vitreous opacities 2) Inner retinal thickening 3) Inner retinal opacification 4) Loss of retinal layer stratification 5) ILMD noted at the posterior pole with a few low intensity hyperreflective dots noted within the ILMD	1 day	1) Ocular massage 2) Anterior chamber paracentesis 3) Oral Acetazolamide 250 mg	PL + ve	Atrophy, thinning and necrosis of the retinal layers ILMD + + Macula hole + +	14
5	15/M	None	RE	1 days	14	PL + ve	1) Retinal whitening 2) Cherry red spot	CRAO	1) Posterior vitreous opacities 2) Inner retinal thickening 3) Inner retinal opacification 4) Loss of retinal layer stratification 5) ILMD noted at the posterior pole with a few low intensity hyperreflective dots and material noted within the ILMD	3 days	1) Ocular massage 2) Anterior chamber paracentesis 3) Oral Acetazolamide 250 mg	PL–ve	Atrophy and thinning of the inner retinal layers with reattachment of ILMD	28

*M* male, *F* female, *CVA* cerebrovascular accident, *RE* right eye, *LE* left eye, *CLA* cilioretinal artery, *PL* perception of light

**Table 2:** Comparison in the clinical and optical coherence tomography (OCT) features between eyes with internal limiting membrane detachment (ILMD) and no ILMD in acute central retinal artery occlusion (CRAO) cases

Variable		Acute CRAO with ILMD (n = 5)	Acute CRAO with no ILMD (n = 23)
Age (years)		34.8 ± 20.51	44.44 ± 20.62
Gender (M: F)		4:1	15:8
Associated systemic disease		1 (20)	13 (57)
Affected eye (RE: LE)		3:2	11:12
Mean duration of symptoms (days) (range)		3.8 (1–7)	3.78 (1–10)
Presenting visual acuity	≤ 3/60	5 (100)	16 (70)
	> 3/60	0 (0)	7 (30)
OCT features at presentation	Posterior vitreous opacities	5 (100)	8 (35)
	Inner retinal thickening	5 (100)	13 (57)
	Inner retinal opacification	4 (80)	20 (87)
	Retinal layer stratification noted	1 (20)	8 (35)
	Cystoid macular edema	0 (0)	5 (22)
	Subretinal fluid	0 (0)	5 (22)
Final visual acuity	≤ 3/60	3 (60)	10 (44)
	> 3/60	0 (0)	11 (56)
OCT features at final visit	Reattachment of ILMD	1 (20)	
	Thinning and atrophy of the inner retinal layers	3 (60)	21 (91)
Total follow-up duration (mean, range)		23.33 (14–28)	60.23 (15–210)

*M* male, *F* female, *RE* right eye, *LE* left eye

## Discussion

The findings in this study suggest that ILMD (5/28 eyes) in cases of acute CRAO are associated with poor presenting visual acuity and more severe signs of retinal hypoperfusion on OCT like inner retinal thickening, inner retinal hyperreflectivity and loss of inner retinal layer stratification. Patients with ILMD have poor final visual acuity and thinning and atrophy or necrosis of the inner retinal layers.

Retinal artery occlusions such as an ophthalmic artery occlusion, central retinal artery occlusion, or, less commonly, a branch retinal artery occlusion can be associated with life-threatening conditions (e.g., carotid occlusive or cardiac valve disease). In patients > 50 years of age, one must additionally suspect giant cell arteritis and if diagnosed should be treated with urgent systemic corticosteroid therapy [7]. Majority of CRAOs occur due to obstruction caused by platelet–fibrin thrombi and emboli as a result of atherosclerotic disease leading to retinal ischemia. These account for over 2/3rd of all CRAO cases [8–10]. Ahn et al. have described the OCT features seen in CRAO during the acute stage and following resolution [4]. The features seen during the acute stage include presence of macular edema, inner retinal thickening, inner retinal hyperreflectivity, inner retinal fluid, loss of inner retinal layer stratification, outer retinal thickening, prominent middle limiting membrane and subretinal fluid. The late features of CRAO on OCT include retinal thinning and foveal photoreceptor defect [4]. The occurrence of what we describe as ILMD has been noted in some previous publications in the figures of their reports [4, 11]. Some authors attribute it to the presence of macular edema and inner retinal fluid [4, 11]. While others believe that there is involvement of the outer retinal layers which could be responsible for this peculiar finding [4, 11, 12]. No clear explanation exists. In this paper, we propose the sequence of events responsible for the occurrence of ILMD in CRAO cases. The explanation is as follows: All tissues or organs can withstand variably short periods of ischemia without producing detectable functional deficits or evidence of injury. Once a critical duration of ischemia is exceeded, which varies by cell type and organ, cell injury and/or death ensues. The extent of cell dysfunction, injury, and/or death is influenced by both the magnitude and the duration of ischemia. During prolonged ischemia, there are several intracellular biochemical and metabolic changes which occur, leading to cell swelling and rupture, and cell death by necrotic, necroptotic, apoptotic, and autophagic mechanisms [13]. Although prompt reperfusion restores the delivery of oxygen and substrates, reperfusion itself appears to have detrimental consequences due to a surge in the generation of reactive oxygen species and proinflammatory neutrophilic infiltration of the ischemic tissues. This concept is termed as reperfusion injury [14, 15]. Thus, following acute CRAO, retinal cell injury and/or death can occur due to severe retinal ischemia and reperfusion injury.

The ILM is the basal lamina of the inner retina that is formed by the footplates of Müller cells [16]. Muller cells are the principal glial cell of the retina. They form architectural support structures stretching radially across the thickness of the retina and are the limits of the retina at the outer and inner limiting membrane, respectively [17]. Müller cell surround blood vessels at the surface of the retina as well as vessels in deeper retinal layers. Following CRAO, there is injury and/or death to the cells of the inner retina depending upon the magnitude of ischemia. With further progression of the tissue injury due to ischemia and reperfusion, there is necrosis and apoptosis of the retinal tissue leading to intraretinal degeneration, permanent necrosis and atrophy. This leaves the ILM unsupported, leading to an ILMD. The hyper reflective dots and material noted in the sub ILM space and in the posterior cortical vitreous could be the proinflammatory neutrophilic infiltrates seen as a response to the reperfusion induced tissue injury. The extensive permanent retinal damage due to atrophy and necrosis is responsible for the poor visual prognosis in these patients. In some cases of CRAO, the ILM detachment was not seen. This could be due to the prompt restoration of retinal reperfusion, thereby allowing the retinal cells to recover immediately without further progression of retinal injury due to ischemia or reperfusion. In 2 cases (case 1 and case 5), where there was reattachment of the ILM to the retinal layers, there was only thinning and atrophy of the inner retinal layers noted without any features of retinal necrosis. The ILMD did not restore in one case where there was necrosis and thinning of the inner retinal tissue.

The major drawbacks of this study are the small number of cases with this peculiar finding and lack of OCT-angiography. The OCT-angiography features in these eyes could have added some valuable information for the development of this finding. Also, further subgroup analysis with statistical tests between the cases of CRAO showing ILMD and those not showing ILMD could have been considered. Unfortunately, the number of eyes with CRAO and ILMD were small. Nonetheless, the results of this study provide newer insights onto the pathomechanism of ILMD in CRAO.

## Conclusion

From the current series of cases, we can conclude that ILMD can occur in acute CRAO due to total retinal artery occlusion and severe retinal ischemia. The presence of ILMD on OCT could be associated with poor visual prognosis. Further work comparing the SD-OCT findings (including ILMD and others) and their relation with OCT-angiography and other multimodal imaging findings needs to be considered in future.

## Data Availability

The datasets used and/or analysed during the current study are available from the corresponding author on reasonable request.

## Abbreviations

CRAO: Central retinal artery occlusion
ILMD: Internal limiting membrane detachment
OCT: Optical coherence tomography

## Declarations

### Research involving human and animal participants

This article does not contain any studies with animals performed by any of the authors.
